# Recognise and Acknowledge Us: Views of Traditional Birth Attendants on Collaboration with Midwives for Maternal Health Care Services

**DOI:** 10.1155/2022/9216500

**Published:** 2022-07-13

**Authors:** Maurine Rofhiwa Musie, Mavis Fhumulani Mulaudzi, Rafiat Anokwuru, Varshika Bhana-Pema

**Affiliations:** Department of Nursing Sciences, Faculty of Health Care Sciences, University of Pretoria, Pretoria 0001, South Africa

## Abstract

**Background:**

Traditional birth attendants have since ancient time provided care to pregnant women. As such, the collaboration between midwives and traditional birth attendant (TBAs) can be an essential effort towards the reduction of the maternal and neonatal mortality and morbidity rate especially in low- and middle-income countries (LMICs). This paper argues that the collaboration between traditional and formal health systems expands the reach and improves outcomes of community health care. The study is aimed at exploring the traditional birth attendant's views on collaboration with midwives for maternal health care services at selected rural communities in South Africa (SA).

**Methods:**

The study was conducted in two rural communities in Tshwane and Johannesburg metropolitan districts from 15 June to 31 October 2021. The study followed the qualitative explorative and descriptive research design. The sampling technique was nonprobability purposive, and snowballing technique was also used to sample the key informants who are the traditional birth attendants also known as traditional healers and who provide maternal health care services in the respective communities. The access to these participants was through the gatekeepers, the Traditional Health Organisation Council (THO) council. Data collection was through semistructured in-depth interviews. Data were analysed thematically through the eight steps of Tesch.

**Results:**

Five main themes were identified which included the recognition of traditional birth attendants as enablers of collaboration, the envisaged value of the collaboration, processes required to foster collaboration, repositioning for new roles, and barriers to collaboration.

**Conclusion:**

The TBAs are ready to collaborate with the formal health care system, and all they require is for their services to maternal health care to be recognised and acknowledged.

## 1. Background

The existence of humankind is attributed to the services of traditional birth attendants (TBAs). With that said, TBAs have a long history as childbirth attendants globally and in many communities in developing countries including South Africa [1]. Traditional birth attendants have played a pivotal role in maternal health care, long before the formalisation and the recognition of midwifery as a distinct area of practice [2]. A TBA is a person who assists the mother throughout the stages of pregnancy and facilitates the childbirth processes. Furthermore, the “TBAs initially acquire their skills by delivering babies themselves or through apprenticeship or from other traditional birth attendants, and they are also referred to as traditional midwives, lay midwives, or comadrona in Southeast Asia” (WHO 1992) [3]. With that said, globally and in African countries, in particular, the World Health Organization [4] has recommended collaborations between health care professionals and traditional TBAs to improve women's access to maternal health care. However, that is not the reality in South Africa; currently, the TBAs are not recognised in the formal health care system.

The recommendation suggested by the WHO and the Declaration of Alma Ata (1978) [5] stipulates that teams needs to be formed between community health workers (CHWs), midwives, physicians, traditional health practitioners (THPs), and traditional birth attendants (TBAs) for the provision of maternal health care services at primary health care level [4, 6, 7]. Currently, there is no collaboration taking place between the two systems, the traditional and western health systems in SA [8]. The current health care system is fragmented, and the pluralistic systems are operating in silos. Studies indicate that approximately 50% of pregnant women in the rural communities (LMICs) consult and prefer traditional birth attendant practice over the western medicine; however, 68% of women across developing regions receive skilled health attendant care [9–12]. The persistence and/or the lack of collaboration between the system seems to undermine the country's ability to progress in terms of the fight against the maternal mortality as indicated by the sustainable developmental goals (SDG) 3 which indicates a global need for maternal mortality rates to be reduced to less than 70 per 100,000 live births by 2030. Furthermore, lack of collaboration may impede on the pregnant women's ability to receive the optimum care needed [13, 14]. The need to progress from such fragmented or merely tolerant health care systems and move towards integrated systems of health service delivery is consequently paramount, which indicates the need for the collaboration of TBAs in the current formal system.

In other African countries such as Zimbabwe, Ghana, and Ethiopia, it is reported that there is inclusion of TBAs in the national health of primary health care and collaborations between midwives and TBAs exist [15, 16]. Benefits of including the TBAs in the formal health care system have been widely reported on [1, 16, 17]. In support, one of the studies done in both Nigeria and Ethiopia indicates that “TBAs are often thought of as the bridge between formal health care and women's cultural beliefs and practices with regard to childbirth” [18, 19]. Literature indicates that the TBAs need to be trained prior to the collaboration in order for them to be included in the health care system. This is very true if we look at countries such as Ghana, where TBAs are trained to identify risks during pregnancy and to refer high-risk women and those likely to have complications to the nearby hospital. Training the TBAs was deemed essential as their competence and prior knowledge are enhanced, and they keep up to date with the current evidence-based practice in midwifery [15]. As such, the authors find it necessary to also explore this approach in SA.

Collaboration between the TBAs and midwives has been considered pivotal in Nigeria and Ghana. In these countries, the TBAs play an important role, as they promote the health and wellbeing of pregnant women and newborns within their communities [9, 20]. The overarching benefit of the collaboration between TBAs and midwives contributes to the global reduction of maternal mortality rates [1, 20, 21]. Currently, the maternal mortality rates in SA remain substantially high at 114 per 1000 births. Thus, it is imperative for SA to integrate the TBAs into the formal health care system. Studies indicate that maternal mortality reduction was achieved by training TBAs on critical roles such as birth preparedness and identification of danger signs during pregnancy. The advantages of linking the TBAs to the health care system had an impact on curbing of perinatal and neonatal deaths and morbidity in various countries [10].

Despite the importance of TBAs in maternal health care in rural and deprived communities in SA, there is paucity of literature regarding the views of TBAs on collaborating with midwives for maternal health care in rural communities. Furthermore, for a successful collaboration, there is a need for a policy to be formed to govern the collaboration [22], although in South Africa the THP Act no. 22 of 2007 has been promulgated and allowed different types of THPs to practice including the TBAs [22–24]. However, the TBA services to maternal health care often go unrecognised in many countries [22]. Thus, the aim of the study is to explore the views of the TBAs with regard to the envisaged collaboration. The collaboration may assist in recognising and acknowledging the crucial role TBAs play in midwifery care.

The study reports on the TBAs' views on collaboration with midwives for maternal primary health care services at selected communities in the Gauteng province of SA. The findings of the study will be useful to the Department of Health (DoH) and other stakeholders involved in formulating policy guidelines for the inclusion of TBAs into the health care system.

## 2. Design and Methods

### 2.1. Research Design

A qualitative explorative and descriptive design [25] was used to explore the views of TBAs on collaboration with midwives for maternal health care in the rural communities of SA. The chosen design was suitable for this study because the researcher gained an in-depth understanding of TBAs' views, as currently, there is a paucity of information regarding this phenomenon.

### 2.2. Research Setting

The research was carried out in two rural communities, in Tshwane and Johannesburg metropolitan districts. The researcher first consulted the THO currently responsible for registering and regulating the TBAs.

### 2.3. Study Population and Sampling Method

The participants for this study comprised both male and female TBAs registered and nonregistered with the THO (Traditional Health Organisation Council); the TBAs were located in the two districts of the Gauteng province of South Africa (SA). The participants were selected using the nonprobability snowballing sampling technique; first, the information regarding the location of participants was received from one of the consulting midwives in the rural community (the identified TBA then acted as an informant to identify other TBAs). The authors identified the gatekeeper of TBAs, who is a representative at the council, who also assisted with linking the researchers to the registered TBA, and the participants were given an option to participate in the study. A total of *n* = 21 TBAs were interviewed.

The inclusion criteria of the study encompassed of the following:

- All the TBAs registered with THO who had experience in the provision of maternal health care
- All TBAs not registered with the THO, who however provide maternal and neonatal health care services within the designated communities
- The TBAs should be willing to participate in the study

### 2.4. Data Collection and Procedure

Data were collected through semistructured individual interviews between June and October 2021. The researcher is a professional advanced midwife trained through western health medicine and was not known by the subject participants. Prior to collecting data, the researcher as an outsider to the community had to obtain permission from the relevant local authorities and the THO. Appointments were scheduled with individual. Information regarding the study was shared verbally through the informed patient leaflet prior the interview process, and the written consent was obtained. The TBAs' the interviews took place at their *Indumba* (sacred healing hut) or in private rooms in TBA homes. On the day of the interview, the TBAs first performed a sacred ancestral ritual by burning incense, to ask permission from the ancestors to participate in the study. The researcher also had to dress appropriately and respectfully (not wear trousers as used to, had to wear a long dress that covers the body to below the knees); this was to respect the African traditional values and beliefs.

The researcher first obtained a written informed consent and informed participants that participation in the interview was voluntary. The researcher utilised an interview guide which contained questions that enquire about the *TBAs' views of collaboration with the midwives for maternal health care*. Follow-up probing questions were also used, to gain an in-depth understanding of the phenomenon under study. Interviews were conducted in the native language of the participants, as the researcher is fluent to the local languages Sepedi and Isizulu. The interviews lasted between 20 and 40 minutes, and field notes were taken during the sessions; COVID-19 measures of precautions, i.e., wearing of protective mask, social distancing, and sanitising of hands, were adhered to throughout. Permission was also sought and received from participants to audio record the interviews. The audio-recorded discussions were transcribed and translated from Sepedi, Isizulu, Sesotho, and Tshivenda to English by a multilinguistic fluent person.

### 2.5. Data Analysis

Data entry and analysis were followed the eight steps of Tesch thematic analysis [26]. The researcher had audio records which were in the vernacular, and these were sent for translation and transcription to a professional translator. Some interviews were conducted in English, the researcher listened to the audio record, and verbatim transcriptions were done. Each transcript was assigned a unique code to refer to the participants as TBA 1 for anonymity and confidentiality purposes. Then, the researcher along with the coresearchers examines the transcripts to gain general sense of the understanding of the data. Then, the researcher broke down the data in smaller units, by grouping similar information together to generate codes. The same transcripts were given to an independent coder to examine the verbatim transcripts that were generated from the data analysis and to code the data. The independent coder coded the emerging themes and subthemes from the transcripts. Predominant themes and subthemes expressed from the individual narratives were identified and classified according to groups using codes and colours, and thereafter, the units were named; the same process was done by the researcher. The grouped findings are then presented in Table 1. Lastly, the researchers, coresearcher, and the coder met to integrate the coded data and interpret the meaning of the data.

### 2.6. Ethical Consideration

Ethical clearance to conduct the study was sought and granted by the University of Pretoria, Research Ethics Committee, and the Faculty of Health Sciences University of Pretoria for approval before actual commencement of the research (ethical clearance certificate number 597/2020). Permission was also obtained from the traditional health practitioners organisation/council before conducting the study. A study involving human beings must deal with the ethical issues in the effort to protect the participants [25]. The ethical considerations maintained in the study were guided by the Helsinki declaration. Also, the guidelines of the Protection of Personal Information (POPI) act were adhered to, to ensure that confidentiality and anonymity of the TBAs was maintained.

## 3. Findings

### 3.1. Profile of the Participants

The participants were made up of a total of *n* = 21 TBAs of which three (3) are not registered with the THO and eighteen (18) are registered. Most of the participants were between the ages of 50 and 65 years. All the participants are residing in rural communities within the Gauteng province, South Africa. The majority are also regarded as traditional healers with experience in maternal and childcare and have been assisting pregnant women throughout all the stages of pregnancy; two of the TBAs had prophetic calling.

### 3.2. TBAs' Views on Collaboration with Midwives for Maternal Health Care

The following themes as discussed in Table 1 below emerged from the data analysis regarding the views of TBA collaboration with midwives for maternal health care services.

**Theme 1 .**

Recognition as an Enabler for Collaboration.

The TBAs held a strong believe that the first need is to be recognised as counterparts in the health care system before the collaboration can even coexist. The following subtheme emanated from the deliberations on the need for formal recognition by the DoH.

*Subtheme* 1.1. The need for formal recognition and acknowledgement by the Department of Health.

“For long our practice has been existing during the time of our grandmothers our ancestors, it is time that our traditional practice is recognised by the DoH.” TBA 16

“We need to be recognised as traditional birth attendant and that we are child birth attendants and assist the women during the pregnancy.” TBA 9

“The only reason I think we need to be recognised is because we are still trying to ensure that as TBAs we are recognized, so that we can work together with the health practitioners. Currently, in my area, I do work with Simunye clinic with the sister in charge of clinic. Our practice is not yet recognised in the community,. as such we would like them to oversee the process and honestly if they can work with us as we are willing to work with them.” TBA 16

The TBA further indicated that they need recognition even for the other roles they play in maternal health care.

“I need to be included in the WBOT (ward-based outreach teams), or recognised as a home-based carer as I do home visits in the community to support the women after they gave birth.” TBA 6

“We need to be respected and recognised for the knowledge and expertise we have.” TBA 11

Another participant argued that the issue of recognition can be formalised by receiving certificates from the DoH:

“As a TBA with 17 years' experience I can add value to the health care system. Maybe we need certificates for the authorities to allow us see our patients in hospitals and also for recognition, if we are to collaborate. Right now, without any certification there can be problems with hospitals when I go there to attend to my patients.” TBA 17

The TBAs highlighted the importance of working together, to be able to curb the maternal and neonatal mortality rates in SA.

“We can save a lot of lives if we work together. We can reduce the number of deaths on mothers and babies. Women who do not want to go to the clinic do come to us. I had a patient who was HIV and I told her to go to hospital but she refused, if we work together we can save more lives by attending to such cases.” TBA 15

Most of the TBAs reiterated the importance of being recognised by the formal health care systems for the collaboration to work.

**Theme 2 .**

Value for collaboration.

The second main theme that emanated from the findings is on the value of collaboration. The TBAs indicated that they have a strong belief that both the traditional practice and the western medicine have a common goal which is to preserve and save the lives of mothers and babies; thus, the communities will benefit from the collaboration to prevent maternal and neonatal mortality rates (MMR). The following quotes support the subthemes emanated from the main theme:

*Subtheme* 2.1. “If we work together we will save lives.”

“The benefits of collaborating and working together is that we all want to save lives.” TBA 11

“It is because we want to save lives, that we agree to working together and achieve good pregnancy outcomes.” TBA 9

“…for patient wellness, yes we need to work together.” TBA 8

“Yes, there is need for collaboration, because even if we have done homebirth, there is still a need to transfer women to hospital for further management. To make sure both the mother and the baby are in a good in good health. Let's say for example the women have a tear, she'll need attention from the hospital to be repaired at the hospital so that she does not bleed (PPH).” TBA 3

Another participant indicated the need to collaborate so that agreements can be reached on diagnoses and intervention. In collaborations, determination can be done on whether the condition needs divine or medical intervention and said:

“Sometimes, nurses misdiagnose the child conditions like ibala (which is that reddish discoloration mark located at the back of the head also known as rigoni or hlogwana (Which is associated with fontanelle, the medical term of hlogwana is small head). If the ibala moves to the baby's fontanelle then the baby may die. This is a cause of a lot of child mortality that is why we need to work with the clinics so that we can assist in diagnosing traditional illnesses which are not understood in western medicine.” TBA 12

The TBAs also indicated the importance of working together and said:

“…we need to collaborate and work hand in hand with the midwives, so that if they identify a problem during pregnancy that needs attention from a traditional healer then they can refer patient to us. So, we work as a team for the betterment of patients' health. It is a win-win situation, we are able to save lives.” TBA 18

“I give the women medication called mpeta (the name for herbs used for protection Tshivenda), the women used the medicine to wash her body and this prevents evil spirits that can cause harm to her and the baby.” TBA 21

Overall, the TBAs can work with the midwives for the betterment and improvement of maternal health in South Africa, as there are pregnant women who still consult and prefer their services.

*Subtheme* 2.2. Reciprocal knowledge and practice sharing.

This subtheme emanated from the main theme, where the TBAs emphasised that both the traditional and western health practices will benefit from each other by sharing the knowledge. The following quotes support the above statement:

“The benefit of the collaboration is that we will be sharing the knowledge and pregnant women will get the best of both worlds and most problems will be solved.” TBA 8

“There is a need for us to be recognised and we must be included as home-based. We are the ababeletisi (traditional birth attendants in isiZulu) and the government has to ensure sure that our services are recognised we can share the duties and knowledge with midwives.” TBA 6

There was also a negative response indicating that the western medicine does not share knowledge with traditional practice, thus the need to change and reciprocal knowledge sharing:

“…right now, during this interview, we share with you our practices, then you will go with to your western practices and write all the information without acknowledging my contribution and all.” TBA 21

The overall sense gathered is that the TBAs are willing to collaborate and share the information with the midwives as long as it is a two-way process.

**Theme 3 .**

Processes required to foster collaboration.

One of the major themes that emerged from this study is the process required for successful implementation of the collaboration between the TBAs and the midwives. The TBAs felt strongly that before they can collaborate with the midwives, they will need intense training regarding the complications associated with pregnancy.

*Subtheme* 3.1. Training as a prerequisite for the collaboration.

Most of the TBAs indicated that they would need to be trained to be able to enrich the skills they already have on maternal health care and stated:

“There is a need for us traditional birth attendants to receive training through workshops, as this will help us to gain more knowledge on the complications arising during pregnancy.” TBA 15

“I need more knowledge and training on hypertension during pregnancy and other high-risk conditions like diabetes and cervical cancer. From our traditional practice I know I can prepare Imbiza (herbal medicine, but I need understand how Western health manages these conditions.” TBA 14

TBAs volunteered to be part of the cadres for health care workers that conduct home visits for the pregnant women:

“I am happy to be part of the training workshop and I do volunteer to be part of the community workers in my area to do visits for the pregnant women.” TBA 13

“After birth we send the women to the hospital because we do not have pregnancy cards, we wish as traditional birth attendant that we can also have our own traditional maternity cards. But first need training on the maternity cards and what it entails. We also need documentation from the department of health showing that we have received training. It will be nice if we can have records that are tight to the system so that we can be appreciated and recognised and not just refer women to the hospital.” TBA 16

Overall, the TBAs indicated that the training can be in a form of workshops and seminars and can help them to understand the health system and they can implement some of those systems.

*Subtheme* 3.2. Bilateral referral systems.

The TBAs indicated that currently, the referral system is unilateral; they are the ones who refer patients to the hospital after assisting the pregnant women. They feel adamant about the systems and recommend that the referral should be a two-way system. The following quotes highlight the issue regarding the unilateral referrals.

“We have a form already developed from our council, that helps us to refer patients to the clinic. On the referral form we are able to indicate the situation of patient and the complications. However, we don't get referrals from them. There are traditional conditions during pregnancy that clinics can refer to us as traditional birth attendants.” TBA 16

“We need the referral to be bilateral. As it is now it is unilateral. The midwives must refer also refer patients for traditional medicine where necessary.” TBA 9 and TBA 21

“There are some conditions like HIV and lack of blood (known as anemia) which as a traditional birth attendant I am not able to diagnose and manage. I throw the bones and see only the signs person is having but not the condition then I refer women to clinic. However, at hospital they see traditional problems like when the baby is not sitting properly (in breech that is caused by traditional things) but will not refer patients to us. Western medicine performs unnecessary operations that could be prevented, as a traditional healer I will search for answers from ancestors as to what is cause.” TBA 8

The TBAs indicated that they also refer the women with high-risk conditions to the clinic, as explored below:

“I consult with the women in my indumba (sacred healing hut), and as a promoter I have to refer women to clinic to check cholesterol, HIV testing and Bp monitoring. The system is currently one-way and not bilateral which is unfair and shows that our practice is undermined.” TBA 17

**Theme 4 .**

Repositioning for new roles.

This theme explored the identification of new roles that TBAs can play if included in the health care system especially at a primary health care level in line with the WHO's suggestions. The following subthemes which support this main theme are discussed below.

*Subtheme* 4.1. Inclusion in the internal stakeholders of the hospital.

The TBAs vouch for their inclusion and being part of the stakeholders working with the hospitals and clinics. They also indicated their interest in being positioned and included in the mainstream as part of the collaborators.

“I know c-section, is the first thing doctors think about when seeing for example prolonged labour complications, why not consult traditional healer that can indicate what is causing that and how it can be resolved. We need TBAs to be located in the hospital so that they can assist with this situation. Again, another challenge is that after deaths and miscarriages in hospitals we are we have to perform cleansing ceremonies.” TBA 17

Another view from the TBAs was their desire to have their own facilities. They even suggested that government should build traditional hospitals.

“We want to work in the hospitals, where we can help the nurses. But then also if the department builds us traditional hospitals then that can work, because nurses will be able to refer patients to us.” TBA 8

“We are willing to go with the women to hospital, if they can allow us to. Only if they can give us a chance.” TBA 5

*Subtheme* 4.2. Inclusion with the WBOTS (ward-based outreach teams) or as home-based carers.

The TBAs indicated that the new roles they can partake in during the collaboration could include working as home-based carers and the following were stated:

“We need to be recognised as part of the WBOT (ward-based outreach teams), or even home-based carers as we do home visits in our communities to support the women after they gave birth.” TBA6

“If the Department of Health need someone to volunteer at the clinics, I am willing to work. I do not see anything stopping us from working together.” TBA 13

Another participant indicated that they could perform postnatal additional roles as home-based carers especially because they are currently doing that:

“For postnatal I can check the baby for things like *ibala* or *Hlogwana*. During home consultations that where I can help with conditions like *hlogwana* because this is a traditional ailment that babies can die from if not treated.” TBA 14

The indigenous childhood illness called *hlogwana* is known to be accompanied by signs and symptoms of greenish watery stools and sunken and not pulsating fontanelle in which the medical terms can be referred to malnutrition from western medicine. Another illness such as ibala or rigoni (which is the reddish marks at the back of neck of the baby); this illness is often called “Stork” bite/nevus simplex spots. The baby presents to a health facility with related symptoms of stiff neck, eyes open widely, fontanelle not pulsating, and baby crying a lot which can be referred to as meningitis in medical reference as cited by Rikhotso et al. [27]. Often, western medicine suppresses the diarrhoea and treat the symptoms. Some of the infants receiving the care do not improve, further resulting in silent death also known as sudden infant death syndrome (SIDS). Most of TBAs are keen and adamant that they can take new roles and assist with the management of the indigenous childhood illnesses.

*Subtheme* 4.3. Birth companionship.

Most of the TBAs were willing to even accompany the women to the hospital as birth companions only if they are allowed to be at the hospital.

“…like at my village in Rustenburg we travel 35km to the hospital. Some things can happen along the way. That is why I should first see the women, then I can accompany her to the hospital if further support if needs be.” TBA 17

“I am willing to also accompany the women to the hospital as some of the women really trust us (traditional) and want us to go with them to hospital for childbirth.” TBA 20

The additional roles that the TBAs perform besides maternal health care provision and giving of herbal medicine need to be recognised by the formal health care system. Also, new roles can be identified so that the TBAs can play their part in the fight against the maternal mortality rates.

**Theme 5 .**

Barriers to the collaboration between the TBAs and midwives.

The TBAs referred to as gogo/makhulu (as a sign of respect and literally means grandmother/grandfather) as an ancestral calling indicated that there are several factors that act as barriers towards the envisaged collaboration between the TBAs and the midwives. The following barriers were discussed:

*Subtheme* 5.1. Undermining and name calling associated to our practice—they refer to us as “witchcraft.”

The TBAs have indicated that there is a need to educate the health professional on their practice so that they too can acknowledge what they are doing:

“We need to teach nurses the difference between the witchcraft and healing. TBA can run workshops to educate the midwives on our traditional practices.” TBA 14

“We need to be respected as we are sharing a common goal of providing health services to the community.” TBA 11

“They (health professional) call herbal medicines witchcraft, and now are they willing to work with us? What has changed?” TBA 12

The TBAs reported incidents where professional nurses were indifferent to their practice and some are even wondering why now because they are called witches. Although this is the case, they have indicated that they are willing to work with the midwives and to share their knowledge.

*Subtheme* 5.2. Stigmatization as a barrier.

For many years, the THP knowledge and practice have suffered under stigmatization and victimisation. The following TBAs indicate the unpleasant experiences they faced throughout the years.

“They (nurses) do not know anything about our (traditional) practices, yet they have a lot of negative things to say. No one has come to us, and no one is interested to come and know what the traditional birth attendants are doing. The stigma they have is unfounded; they think that all traditional healers do is the sacred dancing. They don't know how we operate and they need to come to us and sit with us and learn our ways.” TBA 16

“The nurses have attitude and they scold the patients that we refer to them.” TBA 14

Overall, TBAs indicated that nurses are discriminating against their patients and practice due to a lack of understanding. And they all agree that it is time that they acknowledge the contribution that TBAs make.

## 4. Discussions

The study sought to explore the views of TBAs regarding collaborations with the midwives in rural communities in South Africa (SA), and there is a paucity of empirical evidence concerning the phenomenon under investigation. The present study deduced that the TBAs feel it is pivotal for the health care system to recognise them as “counterparts.” For many years, their contributions has been overlooked [22]. Literature indicates the importance of recognising the TBAs as the dominant providers of childbirth particularly in rural communities; this is very true for countries such as Ghana where similar studies were conducted [1]. In Ghana, they found that it is imperative for the health care system to recognise TBAs, as these strategies are working to enhance the utilisation of traditional birth attendants as TBAs are the first point of contact to the women during childbirth [1]. Overall, the lack of recognition of the TBAs impacts countries' ability to curb the perinatal and neonatal deaths and morbidities [10].

This reality remains in SA that there are no collaboration existing between the health care professionals and the TBAs, despite the recommendations made by the WHO [4]. The reasons for lack of collaboration noted in the study are from lack of formal registration and regulation of TBAs by the Department of Health. Also, the findings indicate lack of reciprocal knowledge and practice sharing between TBAs and midwives. At the moment, the health care system is fragmented and there are no synergies between the traditional and western practice [28]. The value of collaboration between the health professionals and the TBAs is highlighted in countries like Nigeria which was categorised as one of the countries with a high mortality rate of 576 deaths per 100,000 live births [9]. The country found that the TBAs have great potential to contribute to maternal health and because of their utilisation, the mortality rate has dropped. The South African health care system needs to wake up and start utilising the collaboration opportunities.

In this study, it was deduced that the TBAs can contribute to the health care system by collaborating with health professionals on conditions affecting the neonate's afterbirth like *hlogwana* or *rigoni* which are indigenous conditions that can only be explained though traditional medicine [29]. Africans believe that illnesses are not just physiological but sometimes have spiritual undertone to it. Thus, it was concluded in this study that TBAs can contribute to the health care system, by identifying and diagnosing these conditions that require sacred healing, which in most cases are misdiagnosed within the western health care system. However, we also acknowledge that there is dearth of scientific information on the type of support and remedies that traditional birth attendants provide to women during their pregnancy and childbirth [Aziato and Omenyo,2018]. The TBAs in the study indicated that some of the conditions misdiagnosed by the health care system include the indigenous childhood illness such as *hlogwana* and *rigoni*. The health care system defines these conditions affecting neonates as soft parts on the head, known as the fontanelles, which are fibrous gaps that occur when two cranial bones are juxtaposed or where two or more sutures meet [29]. The TBAs in the study indicated that Western doctors misdiagnose baby conditions and term the condition affecting neonates as dehydration (associated with a sunken fontanelle) and capillary naevus (pigmented or red vascular birthmarks) [30], and it is regarded as one of the causes of neonatal mortality. Often, the child is admitted in the health facilities with rigoni (sunken fontanelle), presenting with signs and symptoms of diarrhoea and vomiting; often, the western system will manage the system, some of the infants receiving the care do not improve, and the condition deteriorates resulting in sudden death. Often, the health professionals will record the infant's death as sudden infant death syndrome (SIDS) of unknown cause (Kinney & Tach,2009), whereas in traditional medicine, *rigoni* is related to traditional cause which is a sex-related infection that pregnant women are exposed to and affects the infant afterbirth resulting in the red spot of the fetal head. The traditional health practitioners use natural herbs to treat *rigoni* for both the mother and the baby. The TBAs further indicated that there are other maternal conditions such as prolonged labour or obstructed labour which may sometimes be caused by evil spirits, also confirmed by local study done by Ngomane and Mulaudzi [31] which they were able to resolve traditionally. A common withheld belief amongst Africans is that in obstructed labour, a woman who has been unfaithful has angered the ancestral gods and must be punished with her labour being obstructed. However, studies indicate that traditional birth attendants lack knowledge on the causes of labour and perform inappropriate management of complications during labour (Maimbolwa, Yomba, et al.).

Hence, the collaboration with the TBAs may assist with the reduction of maternal and neonatal mortality in SA, which is made possible by reduction of the complications that may not be due to physiological changes but also addressing the spiritual undertone of the condition. The TBAs indicated that they are able and willing to assist the health professionals to identify the conditions only if they can recognise and respect their practice. Arguments have been made to indicate the vital need for collaboration between TBAs and midwives. However, the participants in this study indicated their “willingness to collaborate” with the health care system; however, certain processes need to be attended to prior the collaboration. The first most crucial step into the collaboration is the need for training, and this is in line with the WHO's recommendations [4]. Presently, the situation remains that in SA, there is no certification for TBAs and their practice is not approved by the government. Most TBAs have received informal training from other expert TBAs, and although some have registered with the THP council, their practice is still not recognised. Other countries such as Ghana have trained the TBAs in efforts to improve the maternal and neonatal outcomes [15]. The United National Populations fund (UNFPA) and the Safe Motherhood Initiative (SMI) have recognised that the TBAs can provide a pragmatic response to the call raised by the WHO in order to meet the millennium developmental goals (MDG) that are now known as the sustainable developmental goals (SDG) #3 for the reduction of maternal mortality rates (MMRs) [12, 14]. The goal is to train TBAs on issues such as induction of labour, cervical ripening before surgical procedures, and of importance the management of postpartum haemorrhage (PPH) [12]. TBAs can be trained in accredited institutions according to the THPs Act for a period of 12 months and receive certificates of competence on completion on issues of antenatal, intrapartum, and postnatal care. [32, 33].

Studies done reveal the benefit of the identification of new roles that the TBAs can play in their inclusion into the formal health care system. Specifying new roles for TBAs acknowledges their cultural and social acceptability in the community. The participants in this study indicated their willingness to adopt to new roles of being birth companions and/or doulas and accompany the women to the health facilities for childbirth [20]. During companionship, the TBAs act as linkages or referral networks and are able to make sure that women attend the antenatal care (ANC) and postnatal care (PNC) [20]. Additionally, other advantages of companionship by the TBAs offer the women emotional, psychological, cultural, and social support that alleviates any anxiety or preconceived traditional beliefs associated with childbirth [1, 17]. Furthermore, the TBAs' embeddedness in traditional cosmologies, cultural rites, and social protocols means they act as cultural brokers for women who typically blend medical care with traditional care. In some countries such as Pakistan, Bangladesh, and Samoa, TBAs are not only trained as assistants but are also recognised for their tactical skills and knowledge including the use of herbs [12].

The challenges to the collaboration between the TBAs and midwives cannot be disregarded and include discrimination and stigmatization against traditional medicine that was identified in this study as well as in the study conducted in Ghana [34]. The participants in the study reported that the midwives often stigmatize their practice and refer to them as witchcraft. Currently, the stigmatization also acts as a barrier to the collaboration, as these attitudes of the midwives need to be addressed prior to the collaboration. Studies suggest that preconceived ideas and the attitudes that the health professionals' manifest are based on lack of knowledge of traditional health medicines and practices. The lack of knowledge may be attributed to the fact that there is no substantive evidence on the practice of traditional birth attendants. Also, the TBAs added that the midwives need to first understand their practice before they stigmatize them as witches, which entails in-service training midwives on the TBA practice [35]). The participants indicated that there are different traditional health practitioners according to the THP Act (22 of 2007) which is a regulatory framework that governs traditional medicine services and these include sangomas, herbalists, traditional birth attendants, and diviners [35]. The stigmatization of TBAs is not limited to SA, and this can be seen in many other countries where the contribution of the TBAs towards maternal health is overlooked. As indicated in this study and studies conducted in both Nigeria and Ghana TBAs have a contribution to make for their inclusion and/recognition for the betterment of the system.

## 5. Conclusion

It was concluded in the study that TBAs would play an important role if there is collaboration especially in the rural parts of South Africa. The benefits of their inclusion have been attributed to the global reduction of the perinatal and neonatal mortality rates. The overall significance of study is that it is responding to the WHO's call for the collaboration between the TBAs and the midwives. Suggestions made in the literature on ways in which the collaboration may coexist could help in the formulation of policies to regulate the TBA practice and training.

## 6. Implication for Nursing Practice

The findings of the study have implications on the nursing practice; midwives need to acknowledge and recognise the contributions that TBAs play in maternal health care services. Furthermore, the study calls for the formal health care system to collaborate with the TBAs in the fight to reduce the maternal mortality rates in SA. The valuable contributions that TBAs make have to be noted, and necessary structures and policies must be formulated to govern their practice of TBAs as we cannot ignore the fact that women in rural communities consult them.

## Figures and Tables

**Table 1:** Themes and subthemes.

Themes	Subthemes
Theme 1: recognition as an enabler of collaboration	(1.1) The need for formal recognition and acknowledgement by the Department of Health (DoH)
Theme 2: value for collaboration	(2.1) “If we work together we will save lives” (2.2) Reciprocal knowledge and practice sharing
Theme 3: processes required to foster collaboration	(3.1) Training as a prerequisite of collaboration (3.2) Recommendations for bilateral referral systems
Theme 4: repositioning for new roles	(4.1) Inclusion in the internal stakeholders of the hospital (4.2) Inclusion with the WBOTS (ward-based outreach teams) or as home-based carers (4.3) Birth companionship
Theme 5: barriers to the collaboration between the TBAs and midwives	(5.1) Undermining and name calling associated to the TBA practice—they refer to us as “witchcraft” (5.2) Stigmatization as barrier

## Data Availability

The data used to support the findings of the study are available from the corresponding author upon request.

## Abbreviations

TBAs: Traditional birth attendants
HCW: Health care workers
WHO: World Health Organization
LMICs: Low- and middle-income countries
THP: Traditional health practitioners
THO: Traditional Health Organisation
DSI: Department of Science and Innovation
POPI act: Protection of Personal Information
MDGs: Millennium developmental goals
SDGs: Sustainable developmental goals
SMI: Safe Motherhood Initiative
PPH: Postpartum haemorrhage
HIV: Human immunodeficiency virus
SA: South Africa
ANC: Antenatal care
PNC: Postnatal care.

## References

[1] Adatara P., Afaya A., Baku E. A., Salia S. M., Asempah A. (2018). Perspective of traditional birth attendants on their experiences and roles in maternal health care in rural areas of northern Ghana. *International Journal of Reproductive Medicine*.

[2] Hernandez S., Oliveira J. B., Shirazian T. (2017). How a training program is transforming the role of traditional birth attendants from cultural practitioners to unique health-care providers: a community case study in rural Guatemala. *Frontiers in Public Health*.

[3] Fox T. (2019). *There are not enough: the banning of traditional birth attendants in Zambia*.

[4] WH Organization (2015). *WHO Recommendations on Health Promotion Interventions for Maternal and Newborn Health 2015*.

[5] Organization W. H. (1978). *Declaration of Alma-Ata*.

[6] Summerton J. (2006). The incorporation of African traditional health practitioners into the South African health care system. *Acta Academica*.

[7] Petzer K. (2006). Traditional birth attendants, HIV/AIDS and safe delivery in the Eastern Cape, South Africa-evaluation of a training programme. *South African Journal of Obstetrics and Gynaecology*.

[8] Nemutandani S. M., Hendricks S. J., Mulaudzi M. F. (2016). Perceptions and experiences of allopathic health practitioners on collaboration with traditional health practitioners in post-apartheid South Africa. *African Journal of Primary Health Care and Family Medicine*.

[9] Amutah-Onukagha N., Rodriguez M., Opara I. (2017). Progresses and challenges of utilizing traditional birth attendants in maternal and child health in Nigeria. *International Journal of MCH and AIDS*.

[10] Haruna U., Kansanga M. M., Bagah D. A. (2019). Repositioning traditional birth attendants to provide improved maternal healthcare services in rural Ghana. *The International Journal of Health Planning and Management*.

[11] Musie M. R., Peu M. D., Bhana-Pema V. (2019). Factors hindering midwives' utilisation of alternative birth positions during labour in a selected public hospital. *African Journal of Primary Health Care & Family Medicine*.

[12] Lane K., Garrod J. (2016). The return of the traditional birth attendant. *Journal of Global Health*.

[13] le Roux-Kemp A. (2010). A legal perspective on African traditional medicine in South Africa. *Comparative and International Law Journal of Southern Africa*.

[14] World Health Organization (2016). *Time to respond: a report on the global implementation of maternal death surveillance and response*.

[15] Choguya N. Z. (2015). Traditional and skilled birth attendants in Zimbabwe: a situational analysis and some policy considerations. *Journal of Anthropology*.

[16] Ohaja M., Murphy-Lawless J., Dunlea M. (2020). Midwives' views of traditional birth attendants within formal healthcare in Nigeria. *Women and Birth*.

[17] Chi P. C., Urdal H. (2018). The evolving role of traditional birth attendants in maternal health in post-conflict Africa: a qualitative study of Burundi and northern Uganda. *SAGE Open Medicine*.

[18] Garces A., McClure E. M., Espinoza L. (2019). Traditional birth attendants and birth outcomes in low-middle income countries: a review. *Seminars in Perinatology*.

[19] Gurara M., Muyldermans K., Jacquemyn Y., Draulans V. (2020). Traditional birth attendants' roles and homebirth choices in Ethiopia: a qualitative study. *Women and Birth*.

[20] Miller T., Smith H. (2017). Establishing partnership with traditional birth attendants for improved maternal and newborn health: a review of factors influencing implementation. *BMC Pregnancy and Childbirth*.

[21] Reeve M., Onyo P., Nyagero J., Morgan A., Nduba J., Kermode M. (2016). Knowledge, attitudes and practices of traditional birth attendants in pastoralist communities of Laikipia and Samburu counties, Kenya: a cross-sectional survey. *The Pan African Medical Journal*.

[22] DoSa Innovation (2019). *Traditional birth attendant contribution to health care system should not be underplayed*.

[23] Nemutandani M. S. (2016). *A model for collaboration between allopathic and traditional health practitioners in the management of HIV/AIDS and TB patients in Vhembe District, Limpopo Province*.

[24] Zuma T., Wight D., Rochat T., Moshabela M. (2016). The role of traditional health practitioners in rural KwaZulu-Natal, South Africa: generic or mode specific?. *BMC Complementary and Alternative Medicine*.

[25] Polit D., Beck C. (2019). *Resource Manual for Nursing Research: Generating and Assessing Evidence for Nursing Practice*.

[26] Creswell J. W., Creswell J. D. (2017). *Research Design: Qualitative, Quantitative, and Mixed Methods Approaches*.

[27] Rikhotso S. R. (2017). *Indigenous Knowledge of Traditional Health Practitioners in the management of Rigoni: grounded theory approach, [Ph.D. thesis]*.

[28] Mulaudzi F. M. (2001). Synergy between indigenous knowledge systems, modern health care system and scientific research-a vision for the 21st century. *Health SA Gesondheid*.

[29] Lekgothoane N., Ross E. (2020). Attitudes of Black South African mothers towards the use of indigenous healing and western medicine in the treatment of newborn infants. *Africa Journal of Nursing and Midwifery*.

[30] Watkins J. (2016). Common skin complaints in neonates. *British Journal of Midwifery*.

[31] Ngomane S., Mulaudzi F. M. (2012). Indigenous beliefs and practices that influence the delayed attendance of antenatal clinics by women in the Bohlabelo district in Limpopo, South Africa. *Midwifery*.

[32] Street R. A., Smith M., Moshabela M., Shezi B., Webster C., Falkenberg T. (2018). Traditional health practitioners and sustainable development: a case study in South Africa. *Public Health*.

[33] Ngunyulu R. N., Mulaudzi F. M., Peu M. D. (2015). Comparison between indigenous and Western postnatal care practices in Mopani District, Limpopo Province, South Africa. *Curationis*.

[34] Krah E., de Kruijf J., Ragno L. (2018). Integrating traditional healers into the health care system: challenges and opportunities in rural northern Ghana. *Journal of Community Health*.

[35] De Lange R. W. (2017). Allopathic and traditional health practitioners: a reply to Nemutandani, Hendricks and Mulaudzi. *African Journal of Primary Health Care and Family Medicine*.

